# Estimation of Wave Condition Number From Pressure Waveform Alone and Its Changes With Advancing Age in Healthy Women and Men

**DOI:** 10.3389/fphys.2020.00313

**Published:** 2020-04-09

**Authors:** Niema M. Pahlevan, Sohrab P. Mazandarani

**Affiliations:** ^1^Department of Aerospace Mechanical Engineering, University of Southern California, Los Angeles, CA, United States; ^2^Division of Cardiovascular Medicine, Department of Medicine, University of Southern California, Los Angeles, CA, United States; ^3^Department of Economics, Geography, and Political Science, Division of Language, Humanity, and Social Science, Riverside City College, Riverside, CA, United States

**Keywords:** wave condition number, arterial wave reflection, optimum cardiovascular function, cardiovascular biomarker, vascular aging

## Abstract

**Introduction:**

The wave condition number (WCN) is a non-dimensional number that determines the state of arterial wave reflections. WCN is equal to HR × L_*eff*_/PWV where HR, L_*eff*_, and PWV are the heart rate, effective length, and pulse wave velocity, respectively. It has been shown that a value of WCN = 0.1 indicates the optimum state of arterial wave reflection in which left ventricle workload is minimized. The pressure wave, flow wave, and PWV are all required to compute WCN, which may limit the potential clinical utility of WCN. The aims of this study are as follows: (1) to assess the feasibility of approximating WCN from the pressure waveform alone (WCN_*Pinf*_), and (2) to provide the proof-of-concept that WCN_*Pinf*_ can capture age related differences in arterial wave reflection among healthy women and men.

**Methods:**

Previously published retrospective data composed of seventeen patients (age 19–54 years; 34.3 ± 9.6) were used to assess the accuracy of WCN_*Pinf*_. The exact value of WCN was computed from PWV (measured by foot-to-foot method), HR, and L_*eff*_. A quarter wavelength relationship with minimum impedance modulus were used to compute L_*eff*_. WCN_*Pinf*_ was calculated using HR and the reflected wave arrival time. Previously published analyses from a healthy subset of the Anglo-Cardiff Collaborative Trial (ACCT) study population were used to investigate if non-invasive WCN_*Pinf*_ captures age related differences in arterial wave reflection among healthy women and men.

**Results:**

A strong correlation (*r* = 0.83, *p*-value <0.0001) between WCN_*Pinf*_ and WCN was observed. The accuracy of WCN_*Pinf*_ was independent from relevant physiological parameters such as PWV, pulse pressure (PP), and HR. Similar changes in WCN_*Pinf*_ with advancing age were observed in both healthy men and healthy women. In young, healthy individuals (women and men) the WCN_*Pinf*_ was around 0.1 (the optimum value), and reduced with aging.

**Conclusion:**

WCN can be approximated from a single pressure waveform and can capture age related arterial wave reflection alteration. These results are clinically significant since WCN can be extracted from a single non-invasive pressure waveform. Future studies will focus on investigating if WCN is associated with risk for onset of cardiovascular disease events.

## Introduction

The cardiovascular system in mammals is based on various optimization criteria (Attinger, 1964; Knight and Wolstenholme, 1971; Milnor, 1979; O’Rourke et al., 1984; Milnor, 1989; Elzinga and Westerhof, 1991). Previous studies have shown that the cardiac dynamics and vasculature characteristics of mammals follow certain allometric laws (Adolph, 1949; Holt et al., 1981; Li and Noordergraaf, 1991; Westerhof and Elzinga, 1991; Li, 1995). Several cardiovascular characteristics are invariant regardless of mammalian size. These size invariant characteristics include mean blood velocity in the ascending aorta (Holt et al., 1981), the product of the heart rate (HR) and the arterial decay time (Westerhof and Elzinga, 1991), the normalized input impedance (Westerhof and Elzinga, 1991), the pulse wave velocity (PWV) (Milnor, 1979, 1989), the reflection coefficient (Li and Noordergraaf, 1991), the product of the propagation constant and the aortic length (Li and Noordergraaf, 1991), and the recently proposed wave condition number (WCN) (Pahlevan and Gharib, 2014).

Pahlevan and Gharib demonstrated the existence of a non-dimensional number, known as the WCN, that determines the optimum arterial wave state in which the left ventricular (LV) workload is minimized in mammalian cardiovascular systems (Pahlevan and Gharib, 2014). Using a series of *in vitro* hemodynamic studies, published hemodynamics data on various mammalian species, and allometry analysis, they have shown that a value of WCN = 0.1 indicates the optimum state of arterial wave reflection in the mammalian systemic circulation. Furthermore, their analysis confirms that this optimum value of the WCN remains constant (0.1) at various levels of aortic stiffness, and is universal among all mammals regardless of size (Pahlevan and Gharib, 2014). Yigit and Pekkan analytically derived a set of non-dimensional parameters using the Buckingham Pi theorem that characterizes pulsatile hemodynamics and its energetic cost (Yigit and Pekkan, 2016). Their work also provided a theoretical background for WCN, as they concluded that WCN can be obtained by combining two of the non-dimensional numbers introduced by them(Yigit and Pekkan, 2016).

It is well-known that various cardiovascular diseases (CVDs) alter arterial wave reflections (Safar and O’Rourke, 2006; Nichols et al., 2011; Salvi, 2012). Therefore, it is possible that WCN has prognostic or predictive value for one or more CVDs. Both pressure and flow waves are required to compute WCN exactly (Pahlevan and Gharib, 2014), which may limit the clinical utility of WCN. Therefore, the primary objective of this manuscript is to demonstrate that WCN can be approximated from a single pressure waveform. Pressure waveforms are easily and non-invasively measured using arterial applanation tonometry, an optical smartphone-based handheld device (Armenian et al., 2018; Miller et al., 2020), or even a smartphone by itself (Pahlevan et al., 2017). Therefore, approximating WCN from a single pressure waveform significantly improves its clinical utility. Previously published retrospective data from Murgo et al. (1980) were used to achieve this objective. The second aim of this study is to provide the proof-of-concept that WCN computed from a single non-invasive pressure waveform can capture age related differences in arterial wave reflections among healthy individuals. Previously published average data from McEniery et al. (2005) was used to investigate the second aim.

## Theory and Methods

### Wave Condition Number Theory

In any discipline of physics, wave dynamics in a medium are dominated by three factors: (1) material properties of the medium that define the wave speed, (2) fundamental frequencies of the waves, and (3) interfaces that create wave reflections. Although other wave characteristics such as dispersion or dissipation also contribute to overall wave dynamics, their effects are not dominant in general. Similarly, wave dynamics in the aorta and the arterial system are primarily controlled by (1) pulse wave velocity (PWV; the wave speed), (2) heart rate (HR; the fundamental frequency), and (3) reflection sites. The WCN number combines all reflection sites existent in various forms within the vascular network (e.g., bifurcation, tapering, impedance mismatch, etc.), and considers a hypothetical total reflection site from which the summated reflected waves appear to be reflected. Pahlevan and Gharib (Pahlevan and Gharib, 2014) used these principles and applied a classical dimensional analysis to derive a dimensionless number, called the WCN, as a function of PWV, HR and the distance between the heart and the hypothetical total reflection site. The WCN concept is a systemic view of examining wave reflections, and *does not imply* that the aorta and its complex wave dynamics can be modeled as a straight tube with a single reflection site at the end.

### Wave Condition Number From Impedance Spectrum (WCN_*PQ*_)

Wave condition number is calculated from effective length (*L*_*eff*_), PWV, and HR using the equation (Pahlevan and Gharib, 2014):

(1) $$W\,C\,N=\frac{H\,R.L_{e\,f\,f}}{P\,W\,V}.$$

Here, *L*_*eff*_ is the distance between the heart and a hypothetical reflecting site from which the summated reflected waves appear to return. The WCN computed from Eq. 1 is referred to as WCN_*PQ*_ throughout this manuscript.

Effective length is computed using pressure and flow waves by applying the quarter wavelength relationship (Milnor, 1989):

(2) $$L_{e\,f\,f}=\frac{c}{4\,f_{Z\,m\,i\,n}}$$

Here *c* is the speed of pressure or flow waves (same as the PWV) and *f*_*Zmin*_ is the lowest frequency among all frequencies in which the amplitude of the impedance modulus is minimum. Mathematically speaking:

(3) $$f_{Z\,m\,i\,n}=Minimum\left[f_{i}\right]\left(i=1,2,\ldots \right),$$

Where *f*_*i*_ is defined as:

(4) $${\frac{d\,\left|Z\right|}{d\,f}|}_{f=f_{i}}=0\&{\frac{d^{2}\,\left|Z\right|}{d\,f^{2}}|}_{f=f_{i}}>0.$$

Here |*Z*| is the amplitude of the impedance in the frequency domain, and is computed from pressure and flow harmonics as:

(5) $$\left|Z\right|=\frac{\left|P_{n}\,\left(\omega \right)\right|}{\left|Q_{n}\,\left(\omega \right)\right|},$$

where:

(6) $$P\,\left(t\right)=P_{0}+\sum_{n=1}^{m}\left|P_{n}\,\left(\omega \right)\right|\,e^{i\,\left(n\,\omega_{o}\,t-\varphi_{n}\right)},$$

(7) $$Q\,\left(t\right)=Q_{0}+\sum_{n=1}^{m}\left|Q_{n}\,\left(\omega \right)\right|\,e^{i\,\left(n\,\omega_{o}\,t-\psi_{n}\right)}.$$

Here, $i=\sqrt{-1}$, ω = 2π*f*, and *P*_*0*_, *Q*_*0*_ are the average of pressure and flow over the cardiac cycle, respectively.

### Wave Condition Number From a Single Pressure Waveform (WCN_*Pinf*_)

The time of the inflection point in the pressure waveform has been recognized as an approximation for the reflected wave arrival time (*t*_*arr*_) (Nichols et al., 2011). Assuming a hypothetical single reflection site, and assuming that the average speed of forward waves and reflected waves throughout the arterial system is the same, *t*_*arr*_ will be twice the wave travel time from the heart to this hypothetical single reflection site. Assuming that PWV is time independent throughout the cardiac cycle, *t*_*arr*_ is related to *L*_*eff*_ and PWV using the equation below:

(8) $$\left(1\,/\,2\right)\,t_{a\,r\,r}=\frac{L_{e\,f\,f}}{P\,W\,V}.$$

Substituting Eq. 8 into Eq. 1 gives:

(9) $$W\,C\,N_{P\,i\,n\,f}=\left(1\,/\,2\right)\,t_{a\,r\,r}\cdot H\,R.$$

**FIGURE 1:**
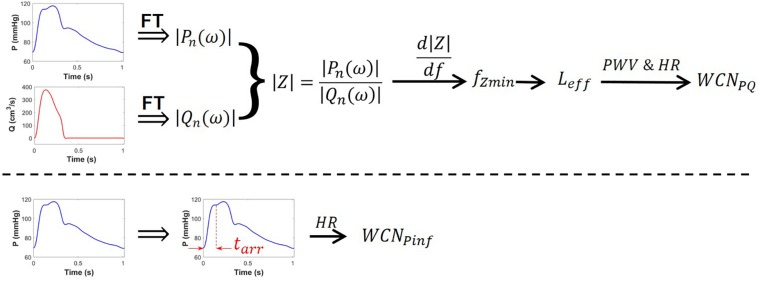
Schematic of the computation of *WCN*_*PQ*_(top row) and *WCN*_*Pinf*_ (bottom row). The pressure wave (blue), the flow wave (red), and PWV are each required to compute *WCN*_*PQ*_. Only the pressure waveform (blue) is needed to compute *WCN*_*Pinf*_. FT is the Fourier transform.

In Eq. 9, HR is expressed in beats-per-second. Figure 1 shows the overall schematic of the computation of *WCN*_*PQ*_ and *WCN*_*Pinf*_.

### Population Characteristics and Hemodynamics Measurements

Previously published retrospective data from Murgo et al. (1980) was used to assess the accuracy of the single pressure waveform evaluation of WCN_*Pinf*_ with respect to WCN computed from Eq. 1 by using the pressure and flow waves and the quarter-wavelength relationship (Eqs 2–7). Data from McEniery et al. (2005) were used to investigate the relationship between non-invasive WCN_*Pinf*_ and aging among healthy individuals.

### Methods for Invasive Evaluation of WCN_*PQ*_ and WCN_*Pinf*_

The database published by Murgo et al. (1980) was used in this study. This database is composed of eighteen patients who underwent right and left heart catheterization for different clinical indications (Murgo et al., 1980). The age range of this cohort was 19–54 years (34.3 ± 9.6). Chest pain was the most common clinical condition in this population. Pressure measurements in the aorta were performed using solid-state pressure sensors (Millar Mikro-Tip, Millar Instruments, Houston, Texas). Electromagnetic flow velocity probes (Carolina Medical Electronics, King, North Carolina 1973–1975; Millar Instruments 1975–1979) were used for flow measurements. PWVs were computed using the foot-to-foot method. The PWV value (needed for the WCN calculation) was not available for one patient, so the database in this manuscript includes the other 17 patients. Further details about hemodynamics measurements and analyses can be found in Murgo et al. (1980).

In seven patients, the inflection point of the pressure waveform occurred before the peak systolic pressure with an augmentation index (AIx) greater than 12% [the so-called type A waveform (Salvi, 2012)]. In seven patients, systolic pressure happened in the late systolic phase following an inflection point with 0 < AIx < 12% [the so-called type B waveform (Salvi, 2012)]. Inflection points occurred after the peak systole in three patients [the so-called Type C waveform (Salvi, 2012)].

### Methods for Non-Invasive WCN_*Pinf*_ and Its Relationship With Age

Reported analyses from McEniery et al. (2005) were used to evaluate non-invasive WCN_*Pinf*_ and investigate its relationship with age in both males and females among healthy populations. The data was a subset of the Anglo-Cardiff Collaborative Trial (ACCT) study population (McEniery et al., 2005). Any individual with clinical history of CVD, evidence of CVD on examination, systolic blood pressure (SBP) ≥140 mmHg and diastolic blood pressure (DBP) ≥90 mmHg, serum cholesterol ≥6.5 mmol/l, renal disease [see (McEniery et al., 2005) for details], and diabetes mellitus were excluded from the healthy subset database. The healthy subset included 4,001 individuals with ages ranging from 18 to 90 years. Aortic pressure waveforms were generated using a validated generalized transfer function (Karamanoglu et al., 1993) applied to radial waveforms measured by a tonometry device (SphygmoCor, AtCor Medical, Sydney, Australia). These synthesized aortic waveforms were then used to identify the inflection point and compute *t*_*arr*_.

### Analysis Method

Bland-Altman analysis (Bland and Altman, 1986) was used to quantify the agreement between WCN_*PQ*_ and WCN_*Pinf*_. The dependency of the error of WCN_*Pinf*_ (defined as the difference between WCN_*PQ*_ and WCN_*Pinf*_) on relevant physiological parameters such as PWV, pulse pressure (PP), and HR was investigated.

The standard deviations (SD) of the non-invasive WCN_*Pinf*_ were approximated from the reported SDs of *t*_*arr*_ and reported SD of the HR and their mean values for each age bracket range assuming that *t*_*arr*_ and HR are independent from each other (Ku, 1966).

## Results

### Accuracy of Single Waveform Evaluation of Wave Condition Number (WCN_*Pinf*_)

**FIGURE 2:**
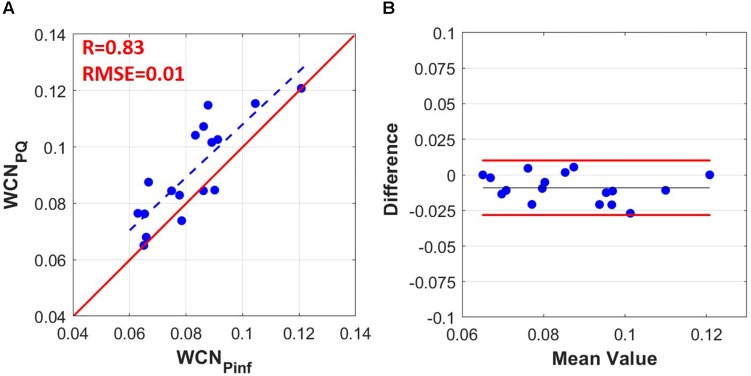
**(A)** WCN_*Pinf*_ computed from the *t*_*arr*_ (time of the inflection point) using Eq. (9) versus WCN_*PQ*_ computed from PQ using Eqs (1–7) (*r* = 0.83, RMSE = 0.01). **(B)** Bland-Altman graph comparing WCN_*Pinf*_ to WCN_*PQ*_. The limit of agreement lines are at +0.010 and –0.028 (–0.009 bias with ± 0.019 limits).

The hemodynamics and the demographics of the study population are shown in Table 1. As illustrated in Figure 2, there is a strong correlation (*r* = 0.83, *p*-value <0.0001) between WCN_*Pinf*_ (WCN computed from the reflected wave arrival time using Eq. 8) and WCN_*PQ*_ calculated from Eq. 1 and, using the effective length computed from Eq. 2.

**TABLE 1:** Study population demographics and hemodynamics.

	**Median**	**Interquartile range**	**Number or mean value (range)**	**Standard deviation**
Age (years)	33	10.25	34.3 (19–54)	9.6
Gender (M/F)	NA	NA	15/2	NA
AoDBP (mmHg)	74	11.75	77 (64–94)	8.4
AoSBP (mmHg)	111	16.25	116.2 (100–146)	11.5
PWV (m/s)	6.59	1.525	6.7 (4.6–9.5)	1.3
HR (bpm)	71	16.25	75.6 (59–110)	13.9
PP (mmHg)	37	9	39.2 (27–73)	11.6

*AoDBP is the aortic diastolic blood pressure, AoSBP is the aortic systolic blood pressure, PWV is the aortic pulse wave velocity, HR is the heart rate, and PP is the aortic pulse pressure.*

**FIGURE 3:**
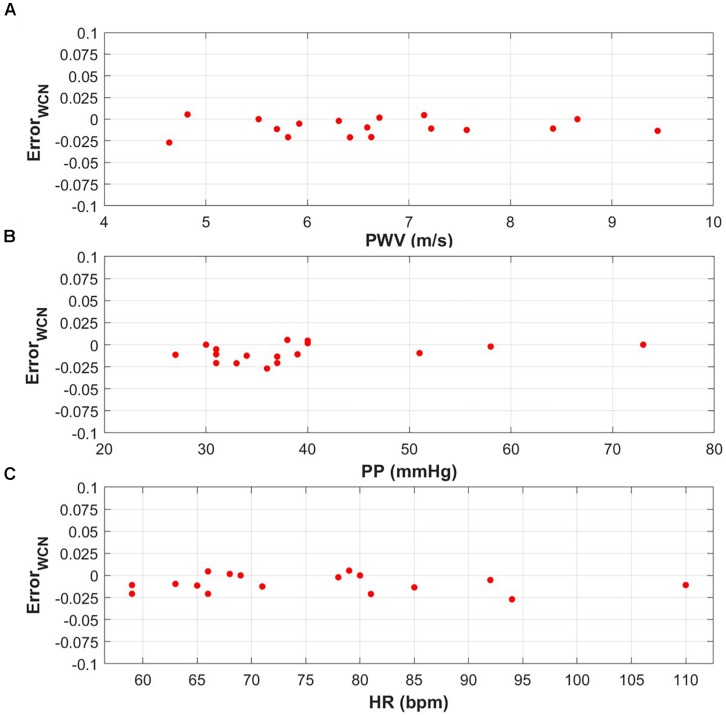
**(A)** WCN_*Pinf*_ error versus a PWV range of 4.64–9.45 m/s (*p*-value = 0.76). **(B)** WCN_*Pinf*_ error versus a PP range of 27–73 mmHg (*p*-value = 0.16). **(C)** WCN_*Pinf*_ error versus a HR range od 59–110 bpm (*p*-value = 0.70).

Figure 3 demonstrates that the WCN_*Pinf*_ error is independent from relevant physiological parameters such as PWV, PP, and HR. There was no significant correlation between WCN_*Pinf*_ error and PWV, PP, and HR with *p*-values of 0.76, 0.16, and 0.70, respectively.

### Non-invasive WCN_*Pinf*_ and Aging in Healthy Population

**FIGURE 4:**
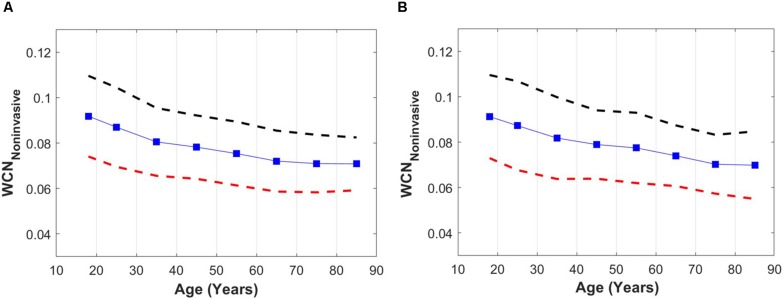
**(A)** WCN_*Pinf*_ versus age among a healthy male population. **(B)** WCN_*Pinf*_ versus age among a healthy female population. Dashed lines are upper (black) and lower (red) standard deviation lines. For better visualization, the *x*-components of data points are set to the mid-points but include the full decade (e.g., *x* = 35 indicates 30–40 years).

The overall declining relationship between WCN_*Pinf*_ and age among healthy populations for both males (Figure 4A) and females (Figure 4B) is demonstrated in Figure 4. The x-components of data points are set to the mid-points but cover the full decade (e.g., *x* = 35 indicates 30–40 years). The dashed bars are the standard deviation lines.

## Discussion

The results of this study indicate that WCN can be approximated from a single pressure waveform measurement. The results also provide a proof-of-concept that non-invasive single waveform WCN (referred to as WCN_*Pinf*_ in this manuscript) can capture age related differences in arterial wave reflection in a healthy population. The results also demonstrate that WCN_*Pinf*_ is approximately 0.1 [the optimum value found by the previous study of Pahlevan and Gharib (2014) for young healthy individuals], and deviates from the optimum value with aging (a reduction from 0.1).

Our results show a strong correlation between WCN_*PQ*_ and WCN_*Pinf*_ (*r* = 0.83, Figure 2A). However, there was an offset of 0.009 (9%) between WCN_*PQ*_ and WCN_*Pinf*_ as illustrated in the Bland-Altman graph of Figure 2B. Prognostic values of WCN and its approximation (WCN_*Pinf*_) will be determined in future clinical studies. On the other hand, the error of WCN_*Pinf*_ did not show statistically significant dependency on hemodynamic parameters related to WCN and overall wave reflection such as PWV, PP, and HR.

Similar changes in WCN with advancing age were observed in both healthy men and healthy women. In young healthy individuals (men and women) the WCN was around 0.1 [the optimum value according to (Pahlevan and Gharib, 2014)], and this reduced with aging as the arterial wave reflections became suboptimal due to vascular aging.

According to the results of Figure 4, the WCN_*Pinf*_ moderately reduces in an aged population. However, the actual decline in the value of WCN among an elderly population is probably more significant since the true value of *t*_*arr*_ in the elderly is over-estimated by the usage of the time of the inflection point. A recent study by Phan et al. (2016), demonstrates that a *t*_*arr*_ computed from the time of the inflection point over-estimates the true value of a *t*_*arr*_ computed from a pressure-flow analysis. Although WCN_*Pinf*_ underestimates the impact of aging on WCN, it reveals the overall trends of aging on WCN among healthy populations. Perhaps WCN computed from other single waveform decomposition methods (Westerhof et al., 2006; Hametner et al., 2013) can provide more accurate single waveform approximations of WCN among healthy aged populations.

Segers et al. (2007) have reported *t*_*arr*_ calculated from the inflection point method and the PQ method (using non-invasive aortic flow and carotid pressure waveforms), in a large middle-aged population (35–56 years old; Asklepios study (Rietzschel et al., 2007) which include 1093 women and 1039 men). Values of WCN_*Pinf*_ computed based on the average values of the reported *t*_*arr*_ and HR (in five-year age intervals reported by Segers et al.) indicate a decrease with age over two decades (35–56 years old) from 0.090 to 0.081 in men and from 0.081 to 0.73 in women. This behavior agree with the results presented in Figure 4. In the latter, the average WCN_*Pinf*_ values and their variations with age are similar in both men and women; however, the average values of WCN_*Pinf*_ computed from the data reported by Segers et al. (2007) are 10% lower in women than in men (0.090 vs 0.081). Furthermore, Baksi et al. (2009) have performed a meta-analysis (64 studies including 13,770 participants with an age range of 4–91 years) to investigate the effect of wave reflections on blood pressure changes that occur with aging. They report a modest but statistically significant (*r* = −0.57, *p* < 0.0001) drop in *t*_*arr*_ with aging. Based on the results reported in Figure 4 of Baksi et al. (2009), *t*_*arr*_ drops from 152 to 120 over 6 decades (20 to 80). Unfortunately, values of HR have not been reported by the authors. Using average HR values for healthy population reported by McEniery et al. (2005), we have computed the corresponding average WCN_*Pinf*_ for the reported data from Baksi et al. (2009): our analysis shows that WCN_*Pinf*_ based on this data drops from 0.92 (20 years old) to 0.62 (80 years old) over six decades. These values are well within the results reported in Figure 4 of this article.

Previous results from a physiologically relevant *in vitro* LV-arterial simulator (Pahlevan and Gharib, 2014) suggest that deviations from a value of WCN = 0.1 increase pulsatile workload on the LV. This workload elevation is more significant at higher arterial stiffnesses (e.g., those occurring with aging). Therefore, a reduction of WCN from 0.1 to 0.07 in a healthy individual may indicate an elevation of the LV pulsatile workload due to suboptimal wave reflections. Future clinical studies are needed to verify if indeed a deviation of WCN away from the optimal 0.1 does in fact increases LV pulsatile workload in a human.

As demonstrated by Westerhof and Westerhof (2018), the uniform tube models inaccurately interpret pressure waveforms and aortic wave travel. However, it must be noted that the WCN concept does not imply in any way that the arterial system is a single tube (as the input impedance is not the same as the actual impedance), and it should not be viewed as an oversimplification. WCN should be considered as a dimensionless number for overall characterization or classification of a wave reflection system. It is comparable to the Reynolds number (Re) in fluid dynamics, which is used for classifications of fluid flow and whose usage is never considered as an oversimplification of boundary layer theory or the Navier-Stokes equations.

Future analyses will focus on investigating if WCN is associated with risk for the onset of cardiovascular disease (CVD) events in large longitudinal cohorts. Such studies will reveal if WCN is a useful addition to standard risk assessment for one or more types of CVDs. Further research can also be focused on the pulmonary vasculature in order to evaluate optimum WCN for the minimization of the workload on the right ventricle (RV). The overall length of pulmonary networks is shorter than the overall length of systemic networks, and the nature of wave reflections at the end of pulmonary vasculature is different than the systemic vasculature (Hollander et al., 2001). These two effects may result in a shorter L_*eff*_. The combining effects of a shorter L_*eff*_ and a lower PWV in the pulmonary artery circulation may produce the same value of optimum WCN (= 0.1) for pulmonary circulation. Although workload on the RV is much lower on than the LV, quantifying the optimum wave reflection can be helpful in patients with right heart failure or patients with pulmonary hypertension (Laskey et al., 1993).

## Limitations

One major limitation of this study is that the database for assessment of the accuracy of WCN_*Pinf*_ did not include any patients older than 54 years. Future studies are needed to verify if the WCN_*Pinf*_ error remains within the same range indicated in this study. Another limitation related to the WCN_*Pinf*_ error is that the left ventricle (LV) ejection fraction (LVEF) values of the patients were not available. LVEF is one of the most relevant cardiovascular parameters that may affect the accuracy of WCN_*Pinf*_. It is noteworthy that there are uncertainties in the assessment of WCN since the absolute accuracy of a WCN assessment depends on the flow and pressure measurement errors, the synchronization between pressure and flow measurements, as well as the sampling frequency of the measurements.

## Conclusion

WCN can be approximated from a single pressure waveform, independently from related hemodynamics indices such as PP, PWV, and HR. This study provides a proof-of-concept that non-invasive single-waveform WCN can capture age-related alterations of arterial wave reflections in a large healthy cohort. However, these changes in WCN with age are not as substantial in healthy populations, possibly limiting its usefulness for healthier individuals. These results are clinically significant since WCN can be extracted from a single non-invasive pressure waveform that is easily acquired using arterial applanation tonometry, a smartphone-based handheld device (Armenian et al., 2018; Miller et al., 2020), or an unmodified iPhone (Pahlevan et al., 2017).

## Data Availability Statement

The data used in this study for the analysis is uploaded as Supplementary Material.

## Ethics Statement

This study uses retrospective previously published data.

## Author Contributions

NP contributed to the conceptualization, the analysis, the writing of the original draft, the revision, and the editing. SM  contributed to the statistical analysis, the revision, the discussion, and the editing.

## Conflict of Interest

The authors declare that the research was conducted in the absence of any commercial or financial relationships that could be construed as a potential conflict of interest.
